# Minimally Invasive Pilonidal Sinus Treatment: A Narrative Review

**DOI:** 10.1515/med-2019-0059

**Published:** 2019-08-07

**Authors:** Nunzio Velotti, Michele Manigrasso, Katia Di Lauro, Enrico Araimo, Federica Calculli, Sara Vertaldi, Pietro Anoldo, Giovanni Aprea, Giuseppe De Simone, Antonio Vitiello, Mario Musella, Marco Milone, Giovanni Domenico De Palma, Francesco Milone, Loredana Maria Sosa Fernandez

**Affiliations:** 1 Department of Advanced Biomedical Sciences, “Federico II” University, Via S. Pansini 5, 80131, Naples, Italy; 2 Department of Clinical Medicine and Surgery. University “Federico II” of Naples, Via S. Pansini 5, 80131 Naples, Italy; 3 Department of Surgical Sciences, University of Campania “Luigi Vanvitelli”, Naples, Italy

**Keywords:** Pilonidal sinus disease, Endoscopic treatment, Minimally invasive surgery

## Abstract

The management of chronic pilonidal disease remains controversial, but recently, new minimal invasive approaches have been proposed. Whereas in the conventional surgical treatment an elliptical wedge of skin and subcutaneous tissue is created to remove the sinus and its lateral tracks, the basis for our new treatment is to create a minimal elliptical wedge of the subcutaneous tissue, including all the inflamed tissue and debris while leaving the overlying skin intact.

The mechanism of an endoscopic approach relies on use of the endoscope without cutaneous tissue damage. Advantages include shorter operative time and time to discharge, which impact resource management in both primary and secondary care: patients undergoing endoscopic technique have a high satisfaction rate, probably due to the low level of postoperative pain and early return to work and daily activities. However, it is mandatory that further studies would analyze surgical approaches to pilonidal sinus disease (PSD) with a consistent and adequate follow-up of at least 5 years. Both sinusectomy and endoscopic approach to PSD were found to be safe and effective compared with conventional techniques. Publishedresults of studies of newer approaches have demonstrated a low short-term complication rate, comparable to conventional surgery results.

## 1 Introduction

Pilonidal sinus disease (PSD) is a common disease of the natal cleft with an incidence of 26 per 100,000 population, affecting predominantly young males adults of working age. The disease usually causes pain and can lead to complications such as abscess formation and recurrent acute or chronic infection [1].

Since its first description in 1883 by Mayo, the management of chronic pilonidal disease remains controversial [2] but recently, following the concept that “less is more”, the new minimal invasive approaches have been proposed: they include sinotomy, sinusectomy, and trephining. Finally, progression of technology and conservative techniques has led to the development of video-assisted and endoscopic pilonidal sinus treatment [3].

## 2 Minimally invasive approach to PSD

### 2.1 Sinusectomy

Sinusectomy consists of circumferential incision of the pilonidal orifices avoiding wide cutaneous margins, and a selective subcutaneous extirpation of the sinus without closure of the wound. Whereas in the conventional surgical treatment an elliptical wedge of skin and subcutaneous tissue is created to remove the sinus and its lateral tracks, the basis for our new treatment is to create a minimal elliptical wedge of the subcutaneous tissue, including all the inflamed tissue and debris, leaving the overlying skin intact.

Minimally invasive strategies for PSD treatment are not unprecedented: in 1983, Bascom et al. described a new technique that combined a local excision and a lateral incision for cavity debridement [4]. The sinusectomy proposed by Soll [5] was introduced as a novel minimal invasive technique for pilonidal sinus to avoid open wide (en bloc) excision; it has demonstrated a low recurrence rate and a fast return to normal daily activities. In 2008, Gips et al [6] proposed a new minimal surgery for pilonidal disease using trephines: in his study on 1358 patients, the author found rates of postoperative infection, secondary bleeding, and early failure of only 1.5%, 0.2%, and 4.4%, respectively. Furthermore, complete healing was observed within 3.4 weeks overall; the recurrence rates after 10 years was 16.2%.

Di Castro and colleagues [7] reviewed a prospectively maintained database with 2347 patients operated using the Gips procedure; they found after a median follow up of 16 months that the recurrence rate was 5.8%, confirming that Gips sinusectomy for the treatment of pilonidal disease is safe and feasible. Similarly, Levinson et al. [8] compared the Gips minimal surgery technique at an Israeli Army clinic with the spectrum of techniques used at public hospitals on 3407 military officers; they reported that patients treated in that clinic had an average of 13 fewer sick leave days. These results confirm that this procedure has a low complication and recurrence rate, allows early return to daily activities, and offers a good cosmetic result.

Elbanna and colleagues [9] also proposed a novel approach: they introduced a sinusectomy accompanied by a thrombin gelatin matrix application as a sealant on 32 patients. Recurrence at 1 year was observed in 2 patients (4%), 96% of patients were satisfied with the procedure, and 92% of patients resumed their daily activities within 3 days.

The Italian group of Neola et al [10] obtained great results with the scarless outpatient ablation of pilonidal sinus (SOAP): among the 31 patients enrolled, after 1 year only 4 patients reported symptoms related to recurrence of the disease. Moreover, patients reported the disappearance of painful symptoms after about 2_._62 days and had been absent from work for only 0_._53 days.

### 2.2 Endoscopic approach

Following the Soll concept that “less is more”, the endoscopic treatment of chronic PSD has been introduced: Milone et al. [11] demonstrated the feasibility of Video-Assisted Ablation of Pilonidal Sinus (VAAPS) and, similarly, Meinero et al. [12] described endoscopic pilonidal sinus treatment (EPSiT). Both VAAPS and EPSiT use a “fistuloscope”, which is inserted into the external opening of the pilonidal sinus: the diagnostic phase identifies the anatomy of the pilonidal sinus and any secondary tracts or abscess cavities by direct vision. The operative phase utilizes forceps for removal of hair within the sinus tract and a monopolar electrode for cautery ablation of the main tract and any secondary tract granulation tissue.

Independently by the name given to the endoscopic approach to PSD, the mechanism of treatment is analogous and focused on the utilization of the endoscope without cutaneous tissue damage. Advantages include shorter operative time and time to discharge with less impact on resource management in both primary and secondary care: patients undergoing an endoscopic technique have a high satisfaction rate, probably due to the low level of postoperative pain and early return to work and daily activities. Initial studies have also shown good healing rates and low recurrence rates. This approach enables the surgeon to directly visualize the sinus tract and any secondary tracts or cavities that may be present: this enables hair removal and homogenous diathermy without missing any areas and avoids unnecessary damage. Another benefit is that, in contrast to open techniques, it does not leave a scar because the surgery is performed via the external opening.

After its introduction, Chia and colleagues [13] in a retrospective study on 9 patients who underwent endoscopic treatment of PSD found a satisfaction rate of 78%, with all patients returning to work less than 5 days postoperatively. In 2016, Meinero consolidated his previous experience with a study on 250 patients with chronic PSD; he found a complete wound healing rate of 94.8%, with a mean complete wound healing time of 26.7 days. [14] Milone and colleagues [15] described a modified VAAPS approach with the use of a prosthetic plug on 4 patients: they introduced a useful minimally invasive scarless technique to treat recurrent pilonidal sinus after failure of other approaches.

Gecim et al. [16] described the safety and effectiveness of crystalized phenol treatment combined with endoscopic pilonidal sinus treatment on 23 patients: there was no or minimal postoperative pain and no primary failure to heal or recurrence was observed at 24 months follow-up. Giarratano et al. [17] found similar results on 77 patients: the median hospital stay was 6.5 hours and the median time to return to work was 5 days, with no major or minor complications, 6 recurrences, and an overall satisfaction rate of 97%. Finally, in 2019, Meinero and colleagues [18] published the results for 122 patients who underwent EPSiT and enrolled in a prospective international multicenter study: these authors found a complete wound healing rate of 95%, a recurrence rate of 5.1%, normal daily activity established on the first postoperative day, and the mean duration before patients returned to work post-operative on day 3.

Minimally invasive technique was performed in acute disease as well: Javed et al. [19] compared traditional incision and drainage with endoscopic treatment for pilonidal abscess and concluded that the endoscopic approach is associated with reduced postoperative morbidity without compromising the adequacy of abscess drainage. Jain et al. [20] also reported their experience with minimally invasive endoscopic technique in acute disease, with encouraging results about postoperative morbidity, recurrence rate, and wound healing.

The stated advantages of conservative surgery have also permeated the guidelines published by the Italian Society of Colorectal Surgery [21] and NICE (National Institute for Health and Care Excellence) guidelines [22] who concluded that minimally invasive techniques should be considered in treatment of pilonidal disease.

### 2.3 Minimally invasive vs conventional treatments

Comparing minimally invasive techniques with others conventional treatments, Emir [23], in a survey of 40 patients treated with sinotomy versus 40 patients treated with surgical excision plus primary closure technique, obtained significant differences in terms of length of hospital stay, time off work, times to sitting on toilet, and walking without pain in favor of minimally invasive approach. A recent meta-analysis by Enriquez-Navascues et al. [24] analyzed results from 4 RCTs comparing a conservative sinusectomy versus radical/en bloc excision with open wound in a total of 153 randomized patients: there were no significant differences in the recurrence rate between the two treatments but a significantly earlier return to work and a lower pain scores following the conservative approach compared with en bloc excision.

Considering an endoscopic approach, instead, Sequeira et al [25] applied endoscopic pilonidal sinus treatment on a pediatric population and compared its results with excision followed by primary closure. Those authors found no differences between groups regarding postoperative complications and complete wound healing; at same time, they recorded a lower recurrence rate in endoscopic group (18.9% vs 21.6%). Successful results were obtained by Milone et al [26], who realized a comparative RCT where 145 patients were randomized to VAAPS or conventional treatment (Bascom’s cleft lift procedure) of pilonidal sinus: in the minimally invasive treatment group, mean time off work was significantly lower, there were fewer infections and patients expressed significantly less pain and higher satisfaction. (Table 1)

**Table 1:** PSD treatment: minimally invasive vs conventional approaches

Author	Patients (n.)	Male : Female	Mean Age (years)	Recurrence (n.)	Pain (VAS)	Satisfaction	Type Surgery	Hospital stay	Follow-up (months)
Emir S. 2014	40	22:18	26.5	0	5.2	1.4	Excision + Primary Closure	1.5 d	12
Emir S. 2014	40	24:16	25.2	0	2.8	2.6	Sinotomy	0.15 d	12
Sequeira JB. 2018	21	16:5	15.9	2	-	-	EPSiT	-	16
Sequeira JB. 2018	63	45:18	16.3	13	-	-	Excision + Primary Closure	-	31
Milone M. 2016	76	60:16	25.5	-	1.5	8.1	VAAPS	-	12
Milone M. 2016	69	54:15	25.7	4	4.5	6.3	Bascom cleft lift	3.2 d	12

## 3 Long-term follow-up

Attention has to be paid to literature data when considering PSD surgery outcomes: few studies have analyzed PSD recurrence rates with a consistent follow-up. It has commonly been assumed that the majority of recurrences occurred in the first year after surgery because in some series, [27] more than 80% of recurrences appeared in the first year postoperative. For this reason, study patients were followed up for short periods and only limited data of longer follow-up times exceeding three years are available [28]. According to Doll, who found that pilonidal sinus may recur up to 22 years after surgery, [29] the short-term follow-up does not allow us to draw definitive conclusions about the recurrence rate of the different surgical techniques. There are only two studies in the literature that confirm this concept: the study of Gips discussed previously and a recent meta-analysis by Milone and colleagues [30]. In that study, the authors demonstrated a long-term follow-up of at least 5 years should be considered the gold standard in pilonidal sinus surgery benchmarking. From this point of view, it is mandatory that further studies analyze PSD surgical approaches with a consistent and adequate follow-up.

## 4 Conclusions

A multitude of treatment for PSD have been proposed, but we are still far from the identification of an ideal approach: the management of chronic pilonidal disease remains controversial. Recurrence rate, infection rate, patients’ pain and satisfaction, time off work, and hospital stay are the focus points around which we need to identify a reliable gold standard. Moreover, it is important to highlight that the general trend of surgery through minimally invasive approaches, has involved also PSD treatment. Both the sinusectomy and endoscopic approaches to PSD were found to be safe and effective compared with conventional techniques. Literature reports demonstrated they have a low short-term complication rate, comparable to conventional surgery results [31]. VAAPS, EPSiT, and video-assisted treatment, are all synonymous of a new technique that has to be considered no more a possibility but a reality. Further randomized, controlled, and high-powered trials with an adequate follow-up, are required to more accurately define the real effectiveness and the eventual side effects of these techniques.
